# One stage anterior sagittal sphincter saving anorectoplasty (ASSSARP) for the repair or rectovestibular fistula: mid and long-term outcome in two tertiary centers

**DOI:** 10.1186/s12887-024-05114-1

**Published:** 2024-10-18

**Authors:** Akram M. Elbatarny, Sherif M. K. Shehata, Mohamed H.M. Ashour, Nezar A. Abo-Halawa

**Affiliations:** 1 Faculty of Medicine, Tanta University, Tanta, Egypt; 2 South Valley Faculty of Medicine, Qena, Egypt

**Keywords:** Rectovestibular fistula, Anterior sagittal, Sphincter-saving, Anorectoplasty, One stage

## Abstract

**Purpose:**

This is a retrospective study of one-stage anterior sagittal sphincter saving anorectoplasty (ASSSARP) for repairing rectovestibular fistula (RVF) including operative details and postoperative complications, functional and cosmetic outcome.

**Patients and methods:**

Records of 41 cases of RVF, managed between April 2010 and September 2019 by one-stage ASSSARP, were reviewed. Preoperative preparation, both early and late postoperative care & complications, hospital stay, and functional & cosmetic outcomes were reported.

**Results:**

The mean age was 6.6 months. Vaginal tear occurred in 5/41 cases, and distal rectal tears in 4/41 cases. Thirteen patients suffered mild superficial wound inflammation; while Skin dehiscence; occurred in five patients. No colostomy or redo was needed. The mean hospital stay was 6.1 days. Mean follow-up was 43.13 months; (Range; 24–100 months). Subclinical anal stricture was detected in six patients. Constipation occurred in 14 cases. Soiling grade I occurred in five patients. Thirty-two patients reached past the age of three years; two of whom showed cough/diarrhea incontinence.

**Conclusion:**

One-stage ASSSARP is safe and gives functional and cosmetic results comparable to other techniques. It provides better access during RVF repair. The avoidance of muscle incision protects against muscle breakdown, if infection sets in, and thus against incontinence. It avoids the morbidity, cost and psychological burden of performing a three-stage repair.

## Background/Purpose

Rectovestibular fistula (RVF) is the most common anorectal malformation (ARM) in females [1–3]. Peña and deVries introduced the PSARP in 1982 with good exposure and dissection. However, for fear of infection which threatens muscle repair and continence, a covering colostomy and a three-stage repair were recommended [4–7]. Okada A. et al. described the anterior sagittal (ASARP) approach in 1992 as one stage [8]. Many authors advocated repairing the ARMs as one stage even in high anomalies when expertise, facilities, patient condition, preoperative preparation, and specialized postoperative care allow [9–17]. This is especially true for RVF, as many surgeons now repair it in one stage [18–25]. Variable techniques for repairing RVF were described, most of which entail dividing the sphincter and suturing it [4, 7, 8]. Postoperative infection carries the risk of muscle breakdown and incontinence. Repair without cutting the muscle obviates this risk and gives good results, when performing one stage. This study was carried out for evaluation of the ASSSARP technique for cases of RVF regarding safety, feasibility, operative details, postoperative complications and functional outcome.

## Patients and methods

**Fig. 1:**
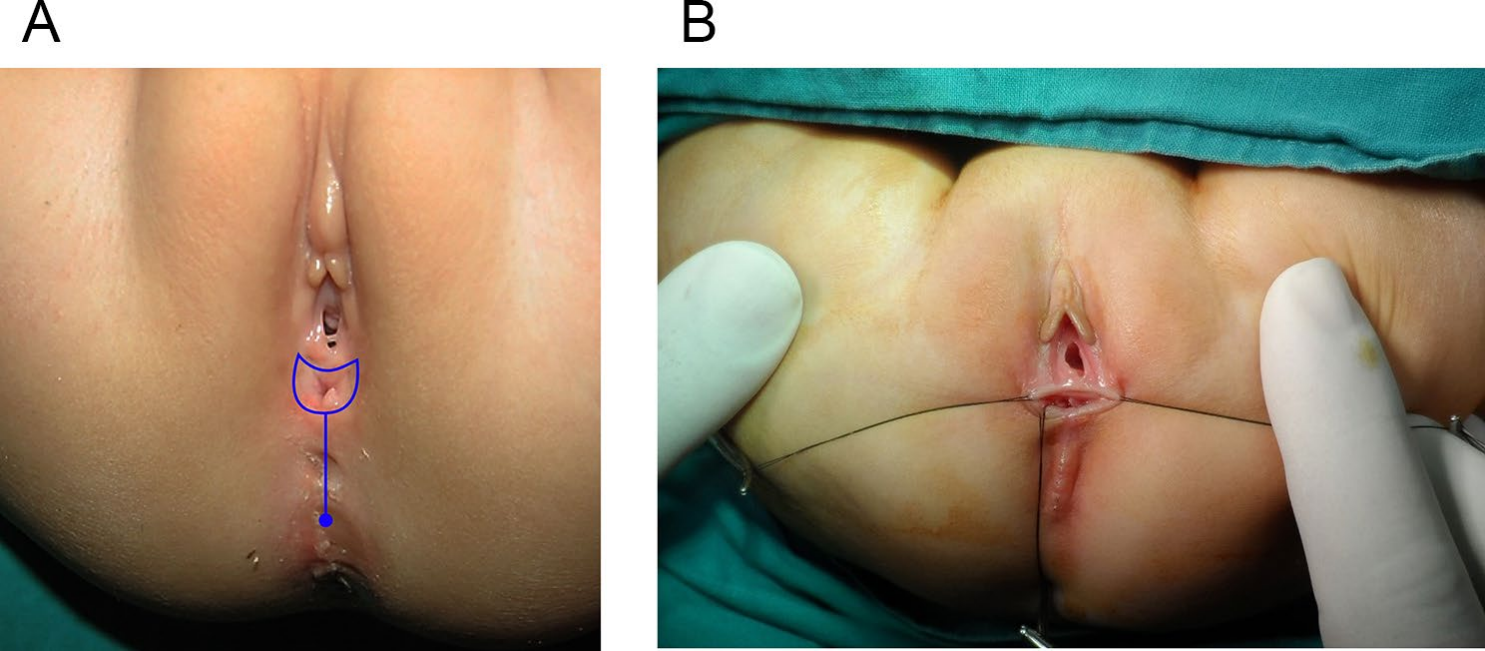
**(a)** Outline of the incision; note the inclining towards the rectum anteriorly. **(b)** Traction Sutures

**Fig. 2:**
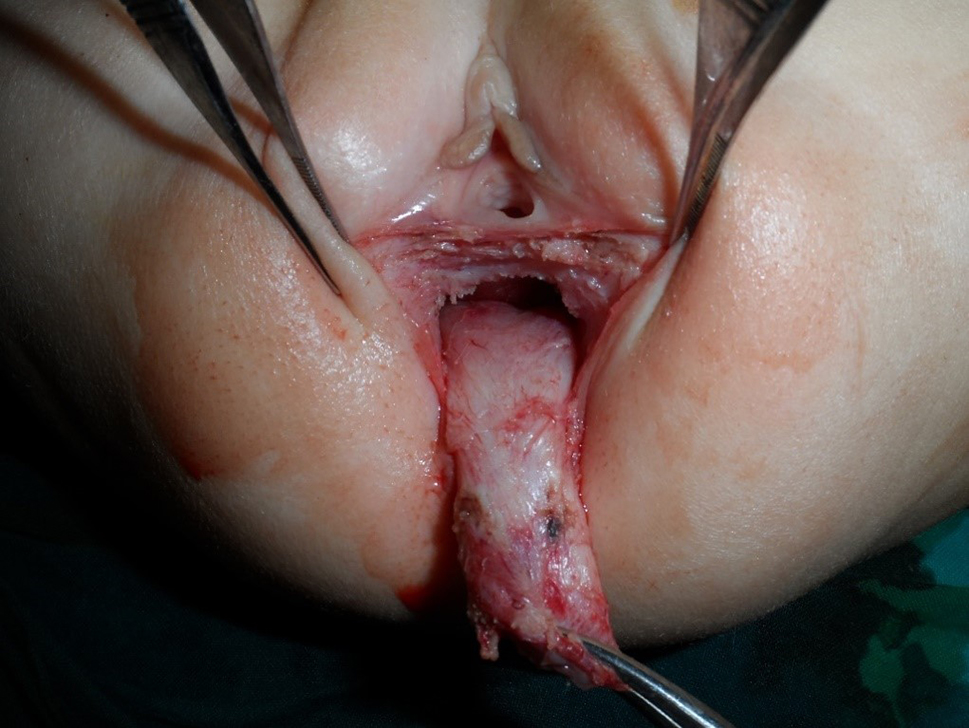
Dissection completed

**Fig. 3:**
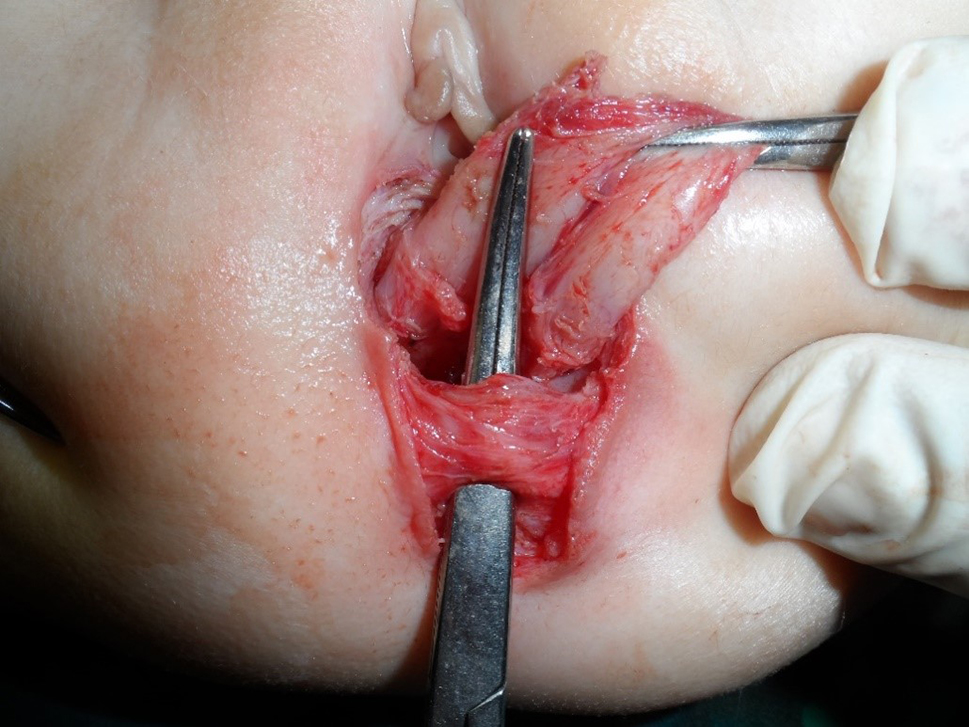
An artery forceps is passed in the center of the sphincter

**Fig. 4:**
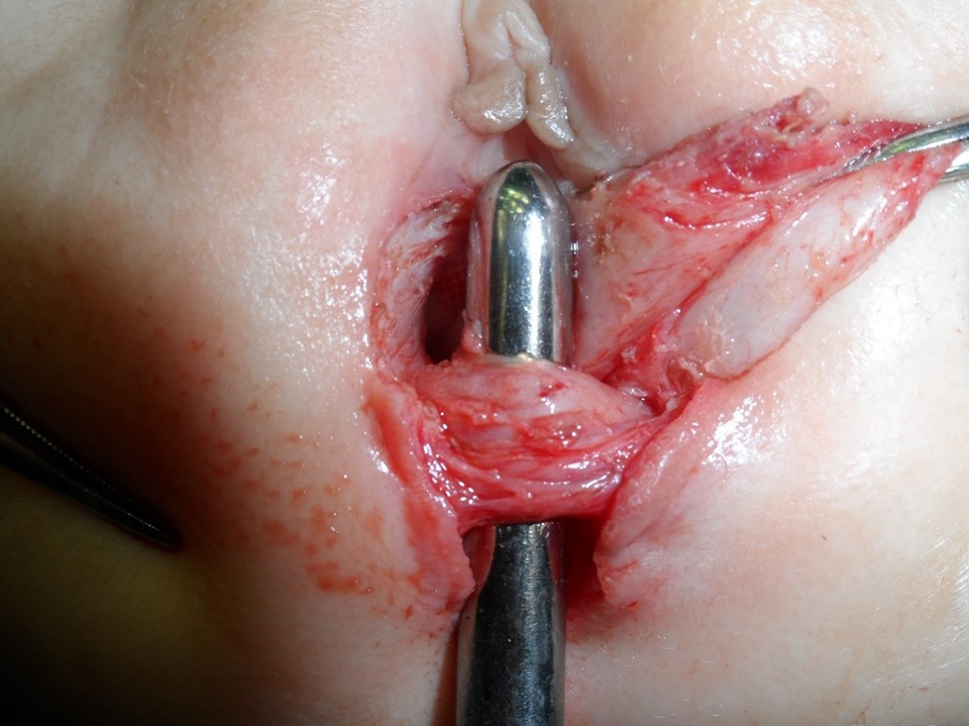
The hole in the sphincter is dilated gradually by Hegars

**Fig. 5:**
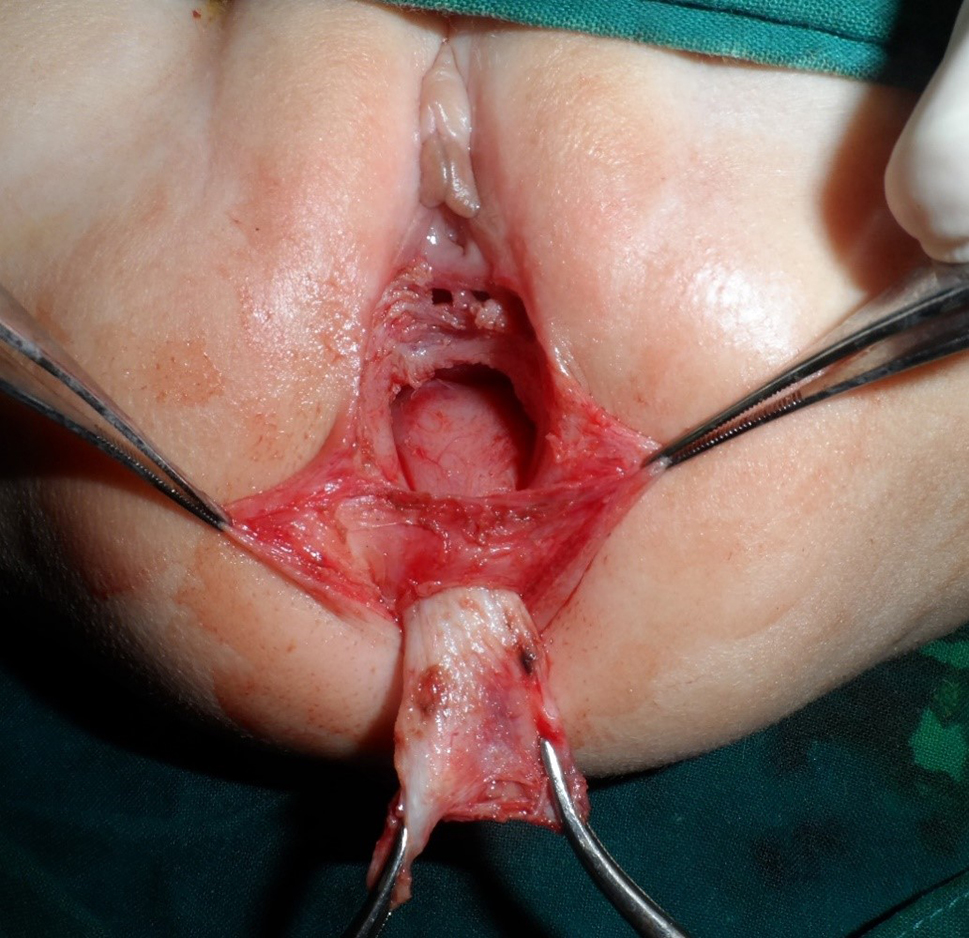
Rectum is passed in the center of the sphincter

**Fig. 6:**
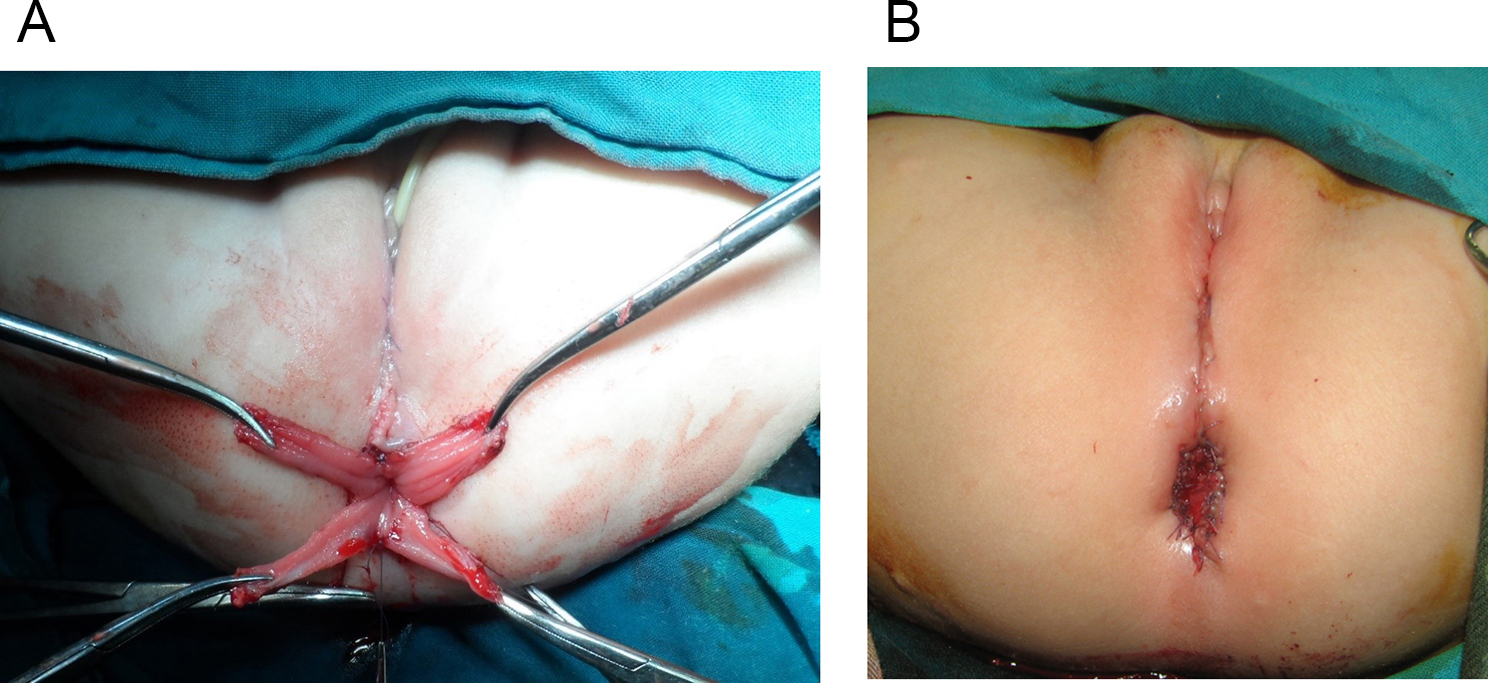
**(a)** Four miles stone sutures. **(b)** Anoplasty completed

This is a retrospective study where the records of 41 cases of RVF repaired and followed up between April 2010 and September 2019 by one stage ASSSARP, in the Pediatric Surgical Unit of Tanta University Hospitals (23 cases), South Valley University hospitals (13 cases), and the private health sector (5 cases), were reviewed regarding the preoperative preparation, operative technique, postoperative care and complications, and functional outcome. Regular incremental dilatation of the fistula was done from diagnosis to repair, to avoid fecal impaction, rectal dilatation, and rectal wall hypertrophy, and to ease preoperative preparation. This was used in a very limited manner in cases operated upon in the neonatal period. One to two day preparation was achieved by rectal washouts using warm saline solution every 3–6 h and giving only clear fluids 24 h before surgery. Oral colistine sulphate is given for one day preoperatively, to avoid using the prophylactic parenteral antibiotics long before surgery, which were given with induction of anesthesia. An NPO was started 4–6 h before anesthesia depending on the age. An antibiotic dose of a combination of 3rd generation cephalosporin, ampicillin/sulbactam and metronidazole, or a combination of linezolid, amikacin, and metronidazole, was given with the induction of anesthesia. The latter combination was the second option, if the first combination was not available, and was avoided, as much as possible, in neonates. The operative technique: the patient is put in the lithotomy position. In infants and young children, this is best achieved by applying the soles of both feet opposed to each other and lightly wrapping them using a gauze tape with cotton padding, and either hanging the gauze tape end to an inverted U-shaped bar (gallows) or stretching and plastering it at the head of the operating table; the latter choice is more suitable for neonates. A Foley catheter is inserted in the urethra. The center of the sphincter is defined and marked; using low power diathermy, in the initial 12 cases in Tanta, and all cases done in South Valley university (13 cases), while Peña muscle stimulator (after it became available) was used in the last 16 cases done in Tanta. Multiple 5/0 traction sutures are taken within the circumference of the fistula. The anterior traction sutures are taken inside the fistula to avoid and preserve the hymen. An incision within the circumference of the fistula is made and continued in the midline posteriorly till the posterior margin of the external sphincter muscle. Again, the anterior half of the incision is made inside the fistula to preserve the hymen; Figure 1. The rectum is dissected starting laterally and posteriorly then proceeding anteriorly to separate the rectovaginal common wall. The first 0.5–1 cm is made submucosally at the expense of the rectal wall, to preserve the hymen, but as we go up, we give advantage to the rectal wall; making great care to keep the rectal wall intact. Accidental vaginal wall tears are immediately repaired with 6/0 Vicryl, after correcting the plain of separation. Dissection is continued until the vagina is completely separated from the rectum and a finger can pass between both structures; Figure 2. The center of the visible external sphincter is again defined by muscle stimulation, then a mosquito is passed through it, and gently opened to dilate the opening in the muscle; Figure 3. The direction of passage of the mosquito should be vertical; to include all fibers of the muscle complex. This is followed by gentle gradual dilatation using sequential Hegar dilators; Figure 4. The rectum is then passed through the opening in the muscle; Figure 5. The perineal body is meticulously reformed using 4/0 Vicryl sutures followed by anoplasty using 5/0 Vicryl sutures. Four initial sutures are used at the 12, 3, 6, & 9 O’clock positions, the excess rectum is trimmed, and spaces between them are filled by sutures placed two mm apart. The skin incision is closed by running 5/0 Vicryl sutures; Figure 6a, b. Oral clear fluids are allowed on the 2nd postoperative (PO) Day and feeding is allowed after the child has passed stools. Meticulous local wound care and cleansing are followed using normal saline, Betadine 10% solution, and fusidic acid ointment. Children were nursed in open diapers to detect and clean any stools immediately, thus avoiding its long contact with the wound. Parenteral preoperative antibiotics were continued for five days; longer for cases of skin infection or dehiscence. In cases of infection antibiotics were changed according to culture and sensitivity from wound swabs. The urinary catheter was removed on day five PO. Anal dilatation program is started on D 14 PO and followed for six months. First size is determined by calibration and then Peña dilatation protocol is followed (1). Both early and late postoperative complications, hospital stay, and functional outcome were reported. Functional outcome was evaluated using Krickenbeck Method for functional assessment; 2005 [26].

## Results

**Fig. 7:**
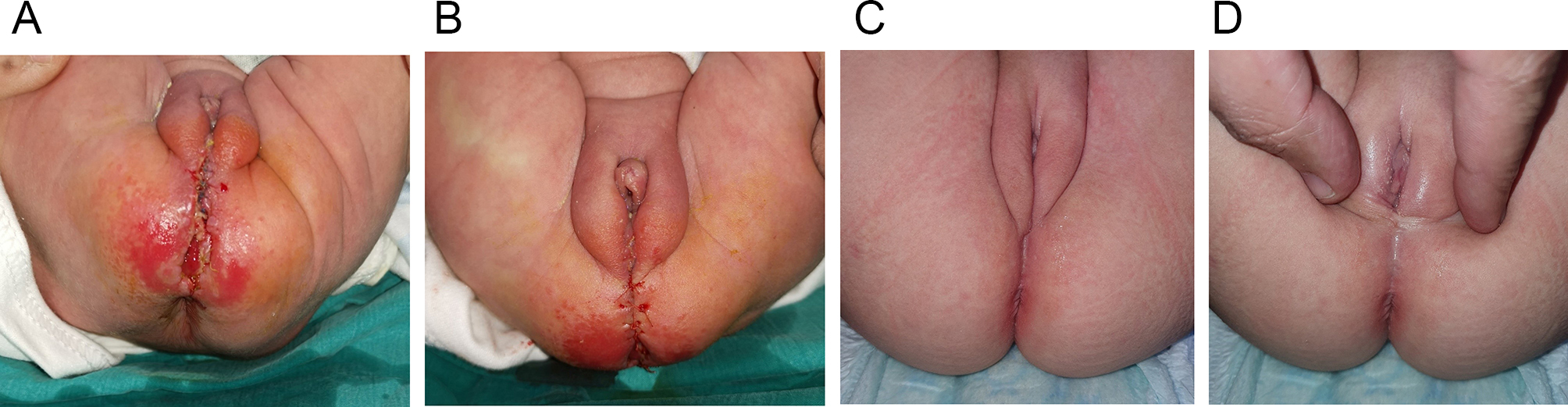
**(a)** Skin dehiscence involving part of the rectal circumference. **(b)** Closed by 4/0 Vicryl sutures at the bedside. **c**,** d.** F/U after 1 year perineum looks nice with an acceptable midline scar

**Fig. 8:**
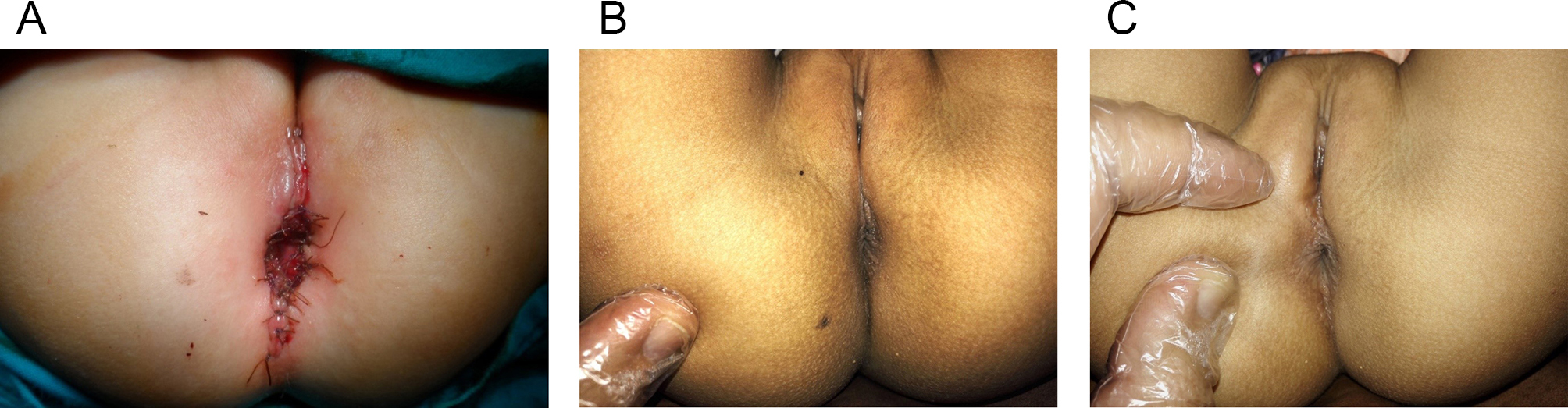
**a.** Operative end view of a 6-month-old girl. (N.B. this was one of the earliest cases where the incision extended behind the neoanus; later no posterior extension of the incision was done. **b**,** c.** Seven years follow up of a patient who had her RVF repaired at the age of 6 months

The medical records of 41 patients with RVF repaired with one stage ASSSARP were reviewed. One to two-day preoperative preparation was done for all cases. The mean age at the time of operation was 6.6 months (Range: 0.23–24 months). The mean operative time was 100.08 min (Range: 75 to 130 min). Vaginal tear occurred in 5/41 cases (12.2%) and was repaired using 6/0 Vicryl. Distal rectal tears occurred in 4 cases (9.8%); the torn rectum was removed with trimming. Mild superficial wound inflammation from contact with stools occurred in 13 patients (31.7%) as well as skin excoriation similar to diaper rash in different degrees affected almost all cases 2–3 days after passage of stools. Skin dehiscence occurred in five patients (12.2%); one case, with separation of part of the neoanus, was resutured by simple sutures as a bedside procedure under LA on day six PO and healed nicely; Figure 7, otherwise, all were managed conservatively. No rescue colostomy was needed. The mean hospital stay was 6.1 days (Range; 5–10 days). The mean follow-up was 43.13 months; (Range; 24–100 months). Anal stricture occurred in six (14,6%). All cases were subclinical strictures detected by calibration during follow-up visits. They were all due to poor compliance with the post-operative dilatation, had no clinical consequences, and were managed by regular home dilatation. Fourteen cases (34.1%) suffered constipation; four were grade I (manageable by changes in diet) and eight were grade II (requires laxatives); managed by diet manipulation and laxatives. Two cases were grade III (Resistant to laxatives and diet), plain abdominal radiographs showed fecal loading, and were managed by disimpaction with enemas before embarking on stimulant laxatives; these two cases also had grade II soiling (soiling every day, no social problem), which improved by treating fecal impaction and constipation. Constipation improved in many patients with long-term follow-up, and patients who were on regular laxatives, turned to take them when needed only. Soiling grade I (occasionally, once or twice per week) occurred in five patients (12.2%), which improved with time in most of them. Soiling grade II occurred in two patients which improved by bowel management as mentioned. Thirty-two patients continued follow-up past the age of 3 years and only two patients (6.25%) showed cough/diarrhea incontinence. The cosmetic appearance was satisfactory to the parents and surgeons in 35 (85.4%) including the one case who was resutured for skin disruption. Figures 7, 8. No redo surgery was needed in any case.

## Discussion

RVF is the most common ARM in girls [1–3]. The sacrum, as well as the sphincters, are usually good. The prognosis for bowel function is excellent when properly treated. Voluntary bowel control is estimated to be about 93% in these girls [1–3, 27]. PSARP was described for the management of these defects covered by a protective colostomy, so the defect was repaired in three stages. This is mainly to avoid the risk of infection and breakdown of the repaired sphincter, which threatens continence [4, 5]. In 1992, Okada A. et al. described the anterior sagittal anorectoplasty (ASARP), where they divided only the anterior part of the muscle complex with the patient in the lithotomy position and sutured it after placing the rectum in the center of the sphincter. However, they kept the legs of their patients immobilized in a cast for 10–12 days and kept them NPO on TPN for the same period. They reported an average hospital stay of four weeks. With this strict and difficult regimen, they had no postoperative wound complications [8].

Colostomy has the great advantage of protecting the repair from the irritating and infectious effects of feces which can lead to wound dehiscence and disruption of the muscle repair (in case the muscles were cut and repaired) and/or rectal retraction. These complications can be serious and might affect the continence of the child. However, colostomy is associated with many complications. Reported rates of colostomy complications range from 28 to 74% [28–34]. The other two functions of colostomy in ARMs; namely GI decompression and imaging of the malformation, are not needed in RVF.

Obviously, three procedures burden the patients and their parents physiologically, psychologically, and economically. Therefore, during the last three decades, many pediatric surgeons reported performing a one-stage correction for intermediate and high ARMs in the newborn period, including male defects, without a protective colostomy with satisfactory results [9–25]; many reported this policy specifically, in RVF and rectoperineal fistula (RPF) [8, 10, 16, 19–25, 35–38].

The fear of postoperative wound infection and disruption can be minimized by good preoperative bowel preparation, antibiotic protection, and meticulous postoperative wound care. However, with repairs entailing cutting and suturing of the sphincter, there is still a chance of muscle breakdown if infection sets in; which threatens future continence. A repair that preserves the muscle, will avoid this complication even if wound infection occurs. Muscle complex preservation (no cutting) was described by some authors; both by ASARP or modified PSARP [19, 20, 39]. Liem N and Quynh T, 2015, thought that preservation of the integrity of the external sphincter could provide better continence and lower rates of constipation as it avoids sphincter scarring [20, 25].

Preoperative bowel preparation in our series was achieved in 24–48 h by both mechanical and chemical preparation. Rectal washouts were the main mechanical way and were done until a clear effluent was retrieved. Different regimens of mechanical preoperative bowel preparation were reported for one-stage repair of RVF over 1–3 days; all include a variable combination of total bowel irrigation and rectal washouts [19–21, 35, 38, 40].

We used parenteral antibiotics on an empiric bases; covering Gram + ve and Gram –ve bacteria as well as anerobes, only within 30 min from the incision which were continued for 5 days (more if infection sets in) postoperatively. Many authors also described the use of prophylactic parenteral antibiotics and continued them for 2–12 days PO [19–22, 35, 38, 41, 42]. Antibiotics are considered prophylactic because this is a threatened wound exposed to fecal contamination early postoperatively. In case wound infection becomes established, they are considered therapeutic and continued as required. This prophylactic antibiotic combination can be looked at as an overtreatment; unless we take into consideration the grave consequences of wound infection in such cases, which might threaten the future continence or lead to deep surgical site infection and sepsis. The ideal and safe combination of antibiotics in such cases can be guided by specific prospective studies based on stool MCS or skin swabs.

In this series, meticulous wound care was followed. The wound was kept open and exposed all the time. This allows immediate detection of any feces, cleansing the wound, and application of local antiseptics. This will avoid any long contact of the feces with the wound and accordingly avoid any chemical irritation or bacterial contamination. Similar wound care regimens were described in many reports [19, 21, 35, 38, 41]. We think this is an essential component of one-stage repair of RVF.

Clear fluids were given 24 h PO, then milk and oral feeds were allowed when the child passed stools. This keeps the wound free from feces until some wound sealing has occurred. Different PO oral allowances have been described. From oral feeding on the 1st PO Day [19, 38], after 24 h [40], to delaying feeding to the 3rd day [37, 41], the 4th day [36], or to the 5th day [21, 22, 42, 43]. More vigorous restriction of postoperative oral intake, for up to two weeks, was described by some authors [8]. We think there is no need to make children suffer for this long period, as the results are not superior with this strict regimen.

Following these measures will avoid any serious surgical site infection (SSI). In spite of these precautions, mild wound affection (infection/inflammation) may occur; which we did have in 43.9% of the patients in the form of mild superficial wound inflammation (13 patients, 31.7%) and superficial skin dehiscence (4 patients, 9.8%), significant skin dehiscence (1 patient; 2.4%). Except for the single case of skin dehiscence, which included part of the circumference of the neoanus and was sutured under LA at the bedside, all were managed conservatively with no serious consequences; thanks to the intact muscle beneath. Mostly, this skin inflammation is the result of chemical contact with stools, as most of these children will have frequent passage of stools in the first few days and almost all of them show perianal skin excoriation similar to napkin dermatitis. Mitul A et al., 2012, used the TFARP technique, where they also preserved the sphincter and the perineal skin [19]. However, from the authors point of view, keeping the perineal skin intact makes reconstruction of the perineal body more difficult by limiting exposure. The option of doing a rescue colostomy, is still there, for patients with serious, deep SSI or sepsis. However, we didn’t need this option, in this series. Varying rates of wound infection and dehiscence have been reported ranging from 7.7 to 30%; all were managed conservatively [19, 21, 35, 40, 41, 44, 45]. Amanollahi O, and Ketabchian S. 2016, in their comparative study on 40 girls with RVF, found 5% in the three-stage repair group vs. 30% wound infection and dehiscence in the one-stage repair group. Despite this high rate of wound complications, wounds were successfully managed - and the authors concluded that single-stage repair is the preferred method considering the drawbacks of the three-stage method [45]. Short S et al. suggested that omitting chemical preoperative preparation, receiving antibiotics for less than 48 h, and early oral postoperative feeding might be factors responsible for wound complications, however, they found no statistically significant risk factors [22]. Waklu et al., 2009 had 2 major wound disruptions (0.4%) after ASARP for RVF, one of them already had a preoperative diverting colostomy. They required a rescue colostomy and revision ASARP after 3–6 months. They stated that neither early oral intake nor defecation are the main factors responsible for wound compromise but rather traumatic dissection, hematoma, or inadequate separation of the rectum from the vagina [38]. Allam A et al., 2016, found no difference in the rate of wound dehiscence among patients repaired with or without a colostomy [42].

We have no rectal retraction even in cases with wound infection or dehiscence. We think this complication can be prevented by complete separation of the rectum from the vagina as high as the peritoneal reflection and good reconstruction of the perineal body. Abouzeid A, in 2015, concluded that in patients with rectoperineal fistula, extended dissection and mobilization of the rectum from the anterior structures (vagina in girls, and bulpospongiosum in boys) decreases wound dehiscence [46]. Mitul A et al., 2012, also performed extensive dissection [19]. Kumar B et al., 2008, agreed with Peña that, anterior migration was due to inadequate separation of the rectum from the vagina [35].

In our study, a subclinical anal stricture was detected in 6 (14 − 6%) patients without apparent clinical consequences. These were detected by calibration during follow-up visits; age matched Hegar dilator cannot be inserted. They were all due to poor parent compliance with the postoperative home anal dilatation protocol. Reported rates of anal stricture from 0 to as high as 26% are reported in the literature [25, 38, 47]. Wehrli L. et al., 2022, in a retrospective study, reported a 0% anal stricture in 84 children with different ARMs. They attributed this rate to preserving adequate blood supply to the bowel, avoiding undue tension on the bowel-to-skin anastomosis, respecting the sphincter limits, and regular postoperative home anal dilations. They also stated that, when the anus is visibly closed at the end of the operation due to the existing sphincter muscle tone, patients will most likely develop a ring-like stricture at the skin level if dilations are not performed [47].

The functional outcome of patients in this series is good and comparable to other series. Constipation is common in those children as stated by Pena [1–3, 27]. Constipation in these patients was reported in different series with a rate of 3.7–47.9% [16, 20–22, 35, 37, 41, 48]. Short S et al., 2018, reported more than 60% constipation in their series [22]. Constipation occurred in 43.9% of our patients and was managed by diet and medications, except for two patients with fecal impaction, which required disimpaction with enemas in addition to laxatives. Those two patients had no anal strictures. With long-term follow-up, constipation improved with age. This observation was also supported by other reports [25, 41, 49]. Mitul et al., 2012, had no cases of constipation in their series [19].

Five (12.2%) children, had grade I soiling; these patients improved with age during follow-up. Two children had grade II soiling but this was found to be due to fecal impaction and improved with bowel management. This is known to occur in normal children with idiopathic constipation and is not related to sphincter weakness. Two (6.25%) out of 32 children, who were > 3 years old during follow-up, showed cough/diarrhea incontinence. Reported rates of soiling and incontinence following one-stage anorectoplasty range from 1.85 to 47.9% [11, 22, 37]. Abdelmohsen S et al. 2022, found that voluntary bowel control, anocutaneous reflex, and anal squeeze response on rectal examination were better in the TSARP and modified ASARP. They proposed that keeping the sphincter intact in those groups vs. division and rejoining the external sphincter muscle in the other techniques, may explain that [25]. The cosmetic appearance also markedly improves with age, and years later, the scar is barely perceived, Figures 7, 8.

The age in our study ranged from 1 week to 2 years, with a mean of 6.6 months. Older ages in other studies, even up to 5-7.5 years, were managed by the same technique [36, 37, 40]. However, from our early experience, we found that in older girls the rectum becomes dilated and its wall hypertrophied. This makes placement of the rectum, in the center of the sphincter, difficult. In such cases, the sphincter may need to be cut and sutured or the rectum tapered, and hence, a colostomy may be needed. We didn’t need rectal tapering in this study. Provided that enough experience is available, the operation is best performed in the neonate as the rectum is not much dilated and can be placed in the center of the sphincter. Theories suggest that neuronal framework for normal bowel and bladder function exists at birth [50, 51]. Neonates are not continent for urine or feces; passage of feces and urine constitutes training that induces neuronal changes [17]. Theoretically, critical time may be lost during which neuronal networks and synapses would have formed if the repair of anorectal anomalies, was deferred beyond 3–4 months. It is supposed that early use of perineal musculature and perianal skin may result in normal or near-normal function [8, 19]. Mitul A et al., 2012, operated on girls from 5 to 28 days old [19]. Liem N & Quynh T 2015, operated on girls aged 3–30 days [20]. Abdul Kadim et al., 2017, found that patients operated upon within the 1st week had no complications compared to a 69% complication rate in children operated on after the 1st week. They attributed this to the easier dissection of virgin tissues and the relatively sterile meconium [21]. Elsherbini et al. and Goyal et al 2020, also advocate neonatal repair of RVF [24, 43]. However, enough surgical experience, anesthetic skills and equipment and perioperative neonatal care must be available to have safe surgery in the neonatal period.

The limited PSARP, where the child is operated in the prone position, has many disadvantages. In this approach, the patient is placed in the prone position which needs special precautions to make the abdomen uncompressed for easing respiration and avoiding hyperextension of the spine. Special padding is also needed to avoid injury to the femoral nerve, especially in older children and longer operations [52]. Anesthetist access to the face and chest is restricted [23, 40]. During the ASARP technique, the child is operated in the supine lithotomy position [8]. This position is more physiologic and more comfortable for the anesthetist, with better access of anesthetists to the chest and face [40]. It also gives a good exposure of the operative field enabling better access of dissection, especially during rectal dissection, and reconstruction of the perineal body [23, 40, 43, 53]. We also support the same view; that this position gives better exposure and access to dissection, especially during separation of the rectum from the vagina, and reconstruction of the perineal body.

During dissection, we aim to preserve the hymen and vagina at the distal one cm, where we go submucosally in the rectal wall as we know this part will usually be discarded, but as we go proximally, we pay more attention not to injure the rectum. In this respect, we had five distal rectal tears (12.2%); the torn rectum was removed with trimming at anoplasty, and four more proximal vaginal tears (9.8%) that were repaired using 6/0 Vicryl with no sequelae. It is also worth noting that most of these injuries happened at the early experience in both centers. Zamir et al., 2008, reported similar concepts [40]. In our series, the hymen was preserved in all cases. This issue is very important for the parents in our culture.

## Conclusion

One-stage ASSSARP is feasible, and safe and gives functional and cosmetic results comparable to other techniques. It provides better access during RVF repair. The avoidance of muscle incision protects against muscle breakdown -if infection sets in- and avoids muscle scars, thus protects against incontinence. One-stage repair also avoids the morbidity, cost and psychological burden of performing three operations.

## Data Availability

The datasets used and/or analysed during the current study are available from the corresponding author on reasonable request.

## Declarations

### Study limitation

the study has the limitation of being retrospective and not comparative.
